# Crossmodal association between visual and acoustic cues in a tortoise (*Testudo hermanni*)

**DOI:** 10.1098/rsbl.2023.0265

**Published:** 2023-07-19

**Authors:** Maria Loconsole, Gionata Stancher, Elisabetta Versace

**Affiliations:** ^1^ School of Biological and Behavioural Sciences, Department of Biological and Experimental Psychology, Queen Mary University of London, London E1 4NS, UK; ^2^ Rovereto Civic Museum Foundation, Borgo Santa Caterina 41, 38068 Rovereto, Trentino, Italy

**Keywords:** crossmodal associations, pitch-size association, *Testudo hermanni*, spontaneous choice, perception, predispositions

## Abstract

Humans spontaneously match information coming from different senses, in what we call crossmodal associations. For instance, high-pitched sounds are preferentially associated with small objects, and low-pitched sounds with larger ones. Although previous studies reported crossmodal associations in mammalian species, evidence for other taxa is scarce, hindering an evolutionary understanding of this phenomenon. Here, we provide evidence of pitch-size correspondence in a reptile, the tortoise *Testudo hermanni*. Tortoises showed a spontaneous preference to associate a small disc (i.e. visual information about size) with a high-pitch sound (i.e. auditory information) and a larger disc to a low-pitched sound. These results suggest that crossmodal associations may be an evolutionary ancient phenomenon, potentially an organizing principle of the vertebrate brain.

## 1. Introduction

In the natural environment, systematic correspondences exist between features encoded in different sensory modalities. For example, because sources of illumination mostly come from above, it is more likely that objects in higher locations are brighter than objects in lower spatial positions [1,2]; similarly, smaller objects resonate with a higher pitch than larger objects and smaller animals are more likely to produce high-pitched sounds compared to larger animals owing to the anatomical structure of their vocal tract [3,4]. Animals can benefit from the ability to create multisensory representations, as not only this ability is associated with enhanced memory [5], but it also confers an advantage in making accurate predictions, reducing uncertainty and allowing the formation of coherent and meaningful representations of objects and events [6,7]. For instance, the pitch-size association can be used to identify the body size of conspecifics [8] or to convey information about resources in the environment [9]. As such, it is likely that different species have evolved expectations (spontaneous predispositions [10,11]) for associations that mirror natural co-occurrences. Previous studies showed that crossmodal associations are consistent across mammals, possibly reflecting a similar prenatal organization and development of the involved neural mechanisms [6,9,12]. Recent data from domestic chicks tested soon after hatching [13,14] show that spontaneous crossmodal associations are available at the onset of life. This suggests that the brain may be evolutionary prepared for crossmodal predictions/associations. In particular, baby chicks spontaneously match luminance and spatial information [13], and visual and tactile information [14]. Few studies have approached the issue of crossmodal correspondences in invertebrates [15,16]. Overall, more studies are needed to understand how widespread are crossmodal correspondences across taxa.

Here, we investigate the presence of crossmodal pitch-size association in a reptile, the tortoise (*Testudo hermanni*). An association between (small and large) visual size and (high and low) acoustic pitch has been reported in humans [4,17–19], chimpanzees [9] and dogs [12]. Tortoises can process auditory information (e.g. they can locate an auditory source to escape a maze [20] and respond to unexpected auditory stimuli, for instance, by retracting their head in the shell [21]). Studies on *T. hermanni* have shown that this species responds to airborne sounds in the range of 10 to 940 Hz [21,22]. Copulating sounds emitted by males can influence females' choice of partners and indicate male quality [22,23]. Although male Hermann tortoises process pitch information, there is no evidence suggesting that they have strong biases for preferring low- versus high-pitch sounds.

We used a two-alternative forced choice task to unveil spontaneous preferences for matching the two dimensions of pitch and size, hypothesizing that we would find evidence of a case of crossmodal correspondence in the tortoise. Based on previous studies in humans [17–19] and dogs [12], and in line with the idea that crossmodal associations correspond to real-world feature associations, we expected to observe a preference to match a high-pitched sound with a small-shape and a low-pitch sound with a larger shape.

## 2. Materials and methods

Detailed materials and methods are reported in the electronic supplementary material, S1.

### (a) Subjects and husbandry

We tested 10 adult male tortoises (*T. hermanni*). Tortoises were housed in groups in an outdoor environment in Sperimentarea (Rovereto Civic Museum Foundation, Italy), where they had free access to fresh water and were regularly fed lettuce and herbs. The experiments were carried out in July–August 2022.

### (b) Experimental arena and training procedures

**Figure 1.**
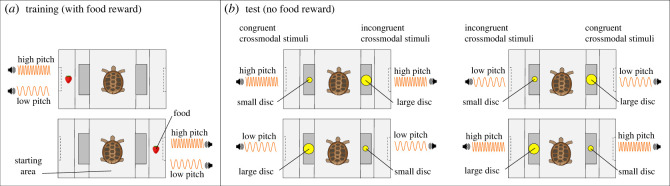
The experimental procedures. At the beginning of each training or testing trial, the tortoise was placed in the centre of the arena (starting area). The tortoise could move from the centre to an adjacent side area (choice area) via the flap door placed on either side. (*a*) In each training trial, a food reward (i.e. a piece of strawberry) was placed on the side area by the playing loudspeaker. During training, the tortoise learnt to approach the source of the sound to find the reward. (*b*) At test, tortoises received no reward. Both speakers played the same pitch sound (either high or low). Each flapping door could be identified by a yellow disc placed in its centre, either small or large. We hypothesized a preference for the smaller disc in the high-pitch sound condition and a preference for the larger disc in the low-pitch sound condition.

The experimental arena consisted of a corridor made of wood divided into three separate areas: a central area (30 × 30 cm) used as the animal's starting point and two choice areas (25 × 30 cm) accessible from the central area by a flap door that could be pushed open by the tortoise. On each of the two short sides of the arena, we placed a loudspeaker. Tortoises were trained to follow an auditory cue to locate the area containing a piece of strawberry (⌀ 0.5 cm) (figure 1). The audio track alternated low- and high-pitched sounds (450 and 700 Hz, approx. 65 dB), each lasting 10 s. Tortoises were trained to push the flap door with their foreleg to enter one of the choice areas via a shaping procedure in which the doors were gradually closed until the desired behaviour was learnt. Each subject moved to the next step of the shaping procedure when responding correctly to six consecutive trials, within 3 min per each trial.

### (c) Testing for spontaneous pitch-size association

The test was carried out the day after the subject completed the training. Tortoises were tested in a two-alternatives forced choice task of 24 consecutive trials, where they were allowed to move in the arena and enter either of the two doors. At this stage, no food reward was delivered, to avoid the animals relying on unwanted cues (e.g. olfactory). During the test, both speakers played either the high- or the low-pitched sound (alternated between trials). One flap door presented a larger yellow disc (5 cm diameter, 0.5 cm thickness), and the other door presented a smaller yellow cardboard disc (2.5 cm diameter, 0.5 cm thickness) in the centre (figure 1). The colour and size of the discs were chosen based on previous literature, to ensure discriminability and salience [24]. The high- or low-pitch presentation was counterbalanced between trials (however see the electronic supplementary material, S1). We scored whether the tortoise chose the door with the larger or the smaller disc, considering a choice when the subject crossed the door with its head and at least half of the shell. We predicted that if tortoises use crossmodal associations, they should choose the door with the small circle more often when hearing high-pitched sound, and the door with the large circle when hearing low-pitched sound [12,17].

## 3. Data analysis and results

**Figure 2.**
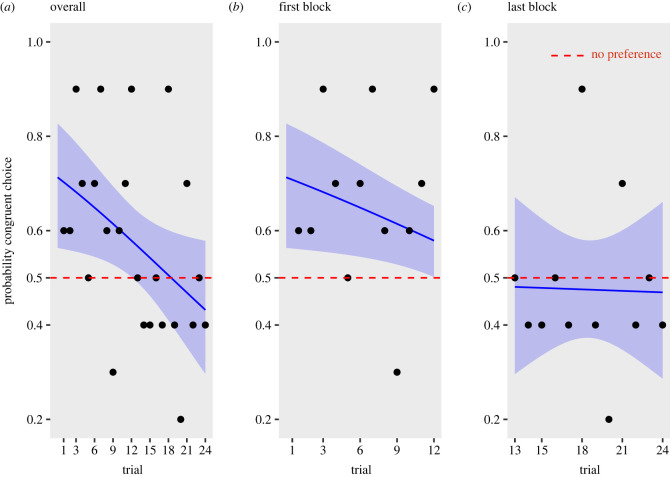
Results. (*a*) Probability of displaying the hypothesized crossmodal association among 24 testing trials when considering the door chosen by the subject. Tortoises initially display a crossmodal matching which progressively decreases to reach chance level (i.e. a probability of 0.5, indicated with the red dashed line). (*b*) Probability of displaying the hypothesized crossmodal association in the first 12 trials. Tortoises show a significant preference for congruent crossmodal matching. (*c*) Probability of displaying the hypothesized crossmodal association in the last 12 trials. Tortoises do not show any significant preference. Each point represents group performance; the shaded area represents the confidence interval.

No subjects or trials were excluded from the dataset. We analysed data using R 4.0.2 [25] and plotted figures with ggplot2 [26]. Alpha was set to 0.05. We used a generalized linear mixed-effect model (R package: lme4 [27]) with the dependent variable being binomial, namely either a congruent (i.e. analogous to that reported in mammals [12,17]) or an incongruent crossmodal association between pitch and size. We ran an Akaike information criterion-based model selection to determine the minimum adequate model, including as predictors the pitch (high or low), the position of the larger stimulus (left or right), the testing trial and their interactions. The best model included the effect of the trial variable only. The trial had a significant negative effect (*β* = −0.05; s.e. = 0.02; *z* = −2.55; *p* = 0.01), with a higher choice for congruent combinations at the beginning of the test—starting from about 0.7 in trial 1—that progressively decreases until reaching chance level (figure 2). This is an expected outcome, as animals progressively extinguish responses to unrewarded tests [13,28,29]. Since we found a significant decrease of crossmodal associations with time, we analysed the initial 12 and final 12 trials separately [13,28,29] (figure 2*b,c*). This analysis confirmed a significant crossmodal association in the initial part of the test and no significant association in the second part of the test. *Post hoc* analysis showed that in the first block (i.e. first 12 trials), tortoises showed a significant crossmodal association for the congruent match (prob. = 0.67, s.e. = 0.043, *z* = 3.58, *p* < 0.01), and that in the final block (i.e. last 12 trials), tortoises showed no significant preference (prob. = 0.48, s.e. = 0.05, *z* = −0.55, *p* = 0.58). To control for a possible effect of fatigue, we compared latency between first, intermediate (12th) and last trials. This was defined as the time intercurred between the moment that the tortoise was released in the arena and moment it completed its first step with one of the forelegs. The analysis showed no difference in latency: estimate mean difference (trial 1 versus 24) = 4.92 s, s.e. = 2.58 s, *t* = 1.91, *p* = 0.17; estimate mean difference (trial 1 versus 12) = 4.3 s, s.e. = 2.58 s, *t* = 1.67, *p* = 0.25, estimate mean difference (trial 24 versus 12) = −0.62 s, s.e. = 2.58 s, *t* = −0.24, *p* = 0.97. This suggests that performance was not affected by physical tiredness.

## 4. Discussion

This study investigated crossmodal pitch-size correspondences in adult male tortoises. We observed a spontaneous association between the two sensory dimensions of acoustic pitch (high versus low) and size (small versus large), similar to what was previously reported in humans [17–19,30] and domestic dogs [12]. These results indicate that a reptile could rely on spontaneous associations between perceptual features in different modalities, like mammals do. We suggest that consistency in crossmodal associations across species can reflect the presence of evolutionary-given expectations evolved in response to shared statistical regularities encountered in nature. It is yet to be understood whether the consistency of crossmodal associations across species results from convergent evolution or from common ancestry. Owing to the richness of sensory information in nature, the nervous system might have evolved to spontaneously rely on multisensory representations of stimuli [5,31], conferring an advantage in terms of creating an honest internal representation of the environment and thus adopting the optimal behaviour in response to different situations. For instance, an individual can predict the size of a not yet visible object by hearing the sound it produces [4,9] (see Introduction). For this mechanism to be efficient, however, it also must be flexible and thus subject to extinction or habituation, to account for specific environmental situations that work against the predisposed behaviour [10,11]. Similarly, in our study, we observed that the initial spontaneous association between pitch and size faded after repeated trials. This is unlikely the result of fatigue, as that would have resulted in animals ceasing to respond to the test. Instead, no difference was found in initiating a movement between the first, 12th, and last testing trial and tortoises kept pushing a door for the whole 24 trials with a progressive decrease of the preference for the congruent crossmodal association. A possible alternative interpretation could be that crossmodal associations serve as an initial strategy in novel situations [13]. In other words, when tortoises first encountered the two shapes they used crossmodal associations to predict the most probable source of the sound according to a predisposed internal representation (e.g. a high-pitched sound is more likely to come from smaller objects). However, after multiple experiences of the pitch-size association not being rewarded, tortoises could have stopped relying on the expected association and started behaving at chance level (or even shifting their choice to incongruent stimuli, as suggested by the observed negative trend). Overall, our data provide evidence of pitch-size crossmodal associations in a reptile. This is an essential step in developing our understanding of the evolutionary origins of crossmodal associations, revealing commonalities in the perceptual organization of phylogenetically far-related species. Based on our discovery, further research can determine how crossmodal associations are formed, whether they are spontaneous or learned, and to what extent can be modified in response to individual experiences and exposure to environmental statistical regularities.

## Data Availability

The dataset generated during the experiment is available as the electronic supplementary material, S2 [32].
